# Prognostic implications of left ventricular inward displacement assessed by cardiac magnetic resonance imaging in patients with myocardial infarction

**DOI:** 10.1007/s10554-023-02861-7

**Published:** 2023-05-30

**Authors:** Takeru Nabeta, Maria Chiara Meucci, Jos J.M. Westenberg, Johan HC Reiber, Juhani Knuuti, Pieter van der Bijl, Nina Ajmone Marsan, Jeroen J. Bax

**Affiliations:** 1 Department of Cardiology, Heart Lung Centre, Leiden University Medical Centre, Albinusdreef 2, Leiden, 2300 RC The Netherlands; 2 Department of Cardiovascular Medicine, Fondazione Policlinico Universitario A. Gemelli IRCCS, Rome, Italy; 3 Department of Radiology, Leiden University Medical Centre, Albinusdreef 2, Leiden, 2300 RC The Netherlands; 4 Medis Medical Imaging Systems, Schuttersveld 9, Leiden, 2316 XG The Netherlands; 5 Heart Centre, University of Turku, Turku University Hospital, Kiinamyllynkatu 4-8, Turku, FI-20520 Finland

**Keywords:** Myocardial infarction, Cardiac magnetic resonance imaging, Left ventricular function

## Abstract

Risk stratification of patients with ischemic heart disease (IHD) still depends mainly on the left ventricular ejection fraction (LVEF). LV inward displacement (InD) is a novel parameter of LV systolic function, derived from feature tracking cardiac magnetic resonance (CMR) imaging. We aimed to investigate the prognostic impact of InD in patients with IHD and prior myocardial infarction. A total of 111 patients (mean age 57 ± 10, 86% male) with a history of myocardial infarction who underwent CMR were included. LV InD was quantified by measuring the displacement of endocardially tracked points towards the centreline of the LV during systole with feature tracking CMR. The endpoint was a composite of all-cause mortality, heart failure hospitalization and arrhythmic events. During a median follow-up of 142 (IQR 107–159) months, 31 (27.9%) combined events occurred. Kaplan-Meier analysis demonstrated that patients with LV InD below the study population median value (23.0%) had a significantly lower event-free survival (P < 0.001). LV InD remained independently associated with outcomes (HR 0.90, 95% CI 0.84–0.98, P = 0.010) on multivariate Cox regression analysis. InD also provided incremental prognostic value to LVEF, LV global radial strain and CMR scar burden. LV InD, measured with feature tracking CMR, was independently associated with outcomes in patients with IHD and prior myocardial infarction. LV InD also provided incremental prognostic value, in addition to LVEF and LV global radial strain. LV InD holds promise as a pragmatic imaging biomarker for post-infarct risk stratification.

## Introduction

Despite a decrease in age-standardized mortality rates for cardiovascular death over recent decades, ischemic heart disease (IHD) remains a leading cause of death worldwide [1, 2]. Left ventricular ejection fraction (LVEF) is the most widely used parameter for the assessment of global LV systolic function,[3] providing both prognostic information and guiding management of patients with IHD and myocardial infarction [4, 5]. LVEF, however, is limited in its characterization of systolic function by its dependence on loading conditions and ventricular geometry [6]. Echocardiographic speckle tracking strain analysis is more sensitive than LVEF for the detection of systolic dysfunction,[3] and LV global longitudinal strain (GLS) has been demonstrated to have incremental prognostic value to LVEF in patients with IHD [7]. Cardiac magnetic resonance (CMR) imaging is the reference standard for assessing LV volumes and function, due to its high accuracy and reproducibility [8]. CMR can also provide information on myocardial infarction scar burden which is associated with unfavourable outcomes in patients with IHD [9]. Nowadays, CMR feature tracking (FT) techniques allow the assessment of myocardial deformation from standard cine CMR images [10, 11]. Impaired LVGLS, measured with CMR-FT, has been linked to mortality and cardiovascular events in patients with IHD [12, 13]. GLS has become the dominant deformation parameter used in clinical practice, and while it is a highly sensitive marker of global systolic dysfunction, it has limited regional sensitivity and reflects only longitudinal deformation. LV wall motion has been used to evaluate regional and radial function for more than four decades on X-ray ventriculography, and has also been applied to echocardiography and CMR. In principle, analysis of inward wall motion overcomes the limitations of LVGLS with respect to regional function and radial function, but hitherto it has been based on non-mathematical visual analysis of wall motion only. FT technology allows much more accurate tracking of the endocardial border, resulting in a novel parameter of LV wall motion, namely LV inward displacement (InD). LV InD is defined as the displacement vector from each point of the endocardial border to the LV center. The potential advantage of LV InD is that it could provide a robust and effective measure of regional myocardial motion. [14] The prognostic implications of CMR-FT derived LV InD have not been investigated in patients with IHD and previous infarction. The aim of the current study was, therefore, to characterize LV InD with CMR in individuals with prior myocardial infarction, and to analyse the prognostic impact of this new LV function parameter in comparison to LVEF, LVGLS and LV global radial strain (LVGRS).

## Methods

### Study population

Patients with previous myocardial infarction who underwent clinically indicated late gadolinium contrast-enhanced (LGE)-CMR between 2004 and 2017 were evaluated retrospectively. Individuals who underwent LGE-CMR within 30 days of myocardial infarction or previous surgical LV reconstruction were excluded from the analysis. Baseline demographic and clinical data were collected from the electronic medical record system of the cardiology department (EPD Vision, version 12.5.4, Leiden University Medical Centre, Leiden, The Netherlands). Cardiovascular medications were optimized according to contemporary guidelines and titrated at the discretion of the treating physician [5, 15]. Cardiac resynchronization therapy (CRT) devices and implantable cardioverter-defibrillators (ICD) were inserted during follow-up, according to prevailing guidelines [16–19]. The institutional review board of the Leiden University Medical Centre approved the retrospective analysis of clinically acquired data and waived the need for written patient consent on an individual level.

### Cardiovascular magnetic resonance data acquisition and analysis

CMR was performed on a 1.5-T Gyroscan ACS-NT/Intera MR system or on a 3.0-T Ingenia MR system (Philips Medical Systems, Best, The Netherlands) using retrospective gating. Cine steady-state free precession (SSFP) CMR images were acquired in the long (2-, 3- and 4-chamber views) and short axes of the LV. LGE-CMR images were acquired 15 min after bolus injection of gadolinium diethylenetriamine pentaacetic acid (Magnevist, Schering, Berlin, Germany) (0.15 mmol/kg) with an inversion recovery, three-dimensional, turbo-field echo sequence with parallel imaging. The heart was imaged in one or two breath-holds with short-axis slices at various levels, depending on the size of the heart [19, 20].

**Fig. 1:**
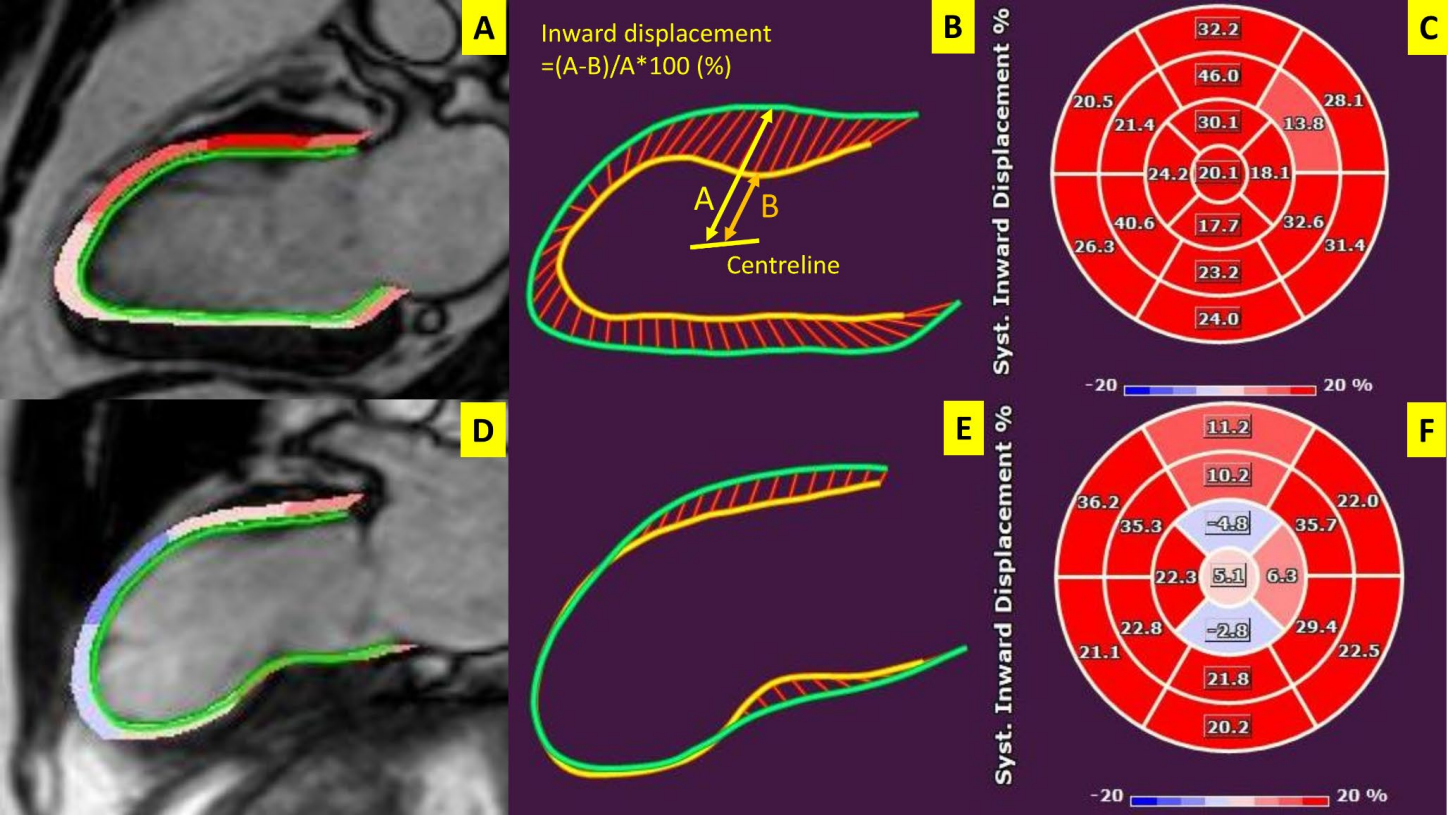
Measurement of left ventricular inward displacement Measurement of left ventricular (LV) inward displacement of two representative cases (normal LV systolic function (**panels A-C**) and a patient with an apical aneurysm (**panels D-F**)). Endocardial and epicardial contours were automatically drawn on long-axis cine images (**panels A, D**). The LV inward displacement of the endocardium was visualized in two dimensions, with the border geometry displayed in end-systole (yellow contour) and in end-diastole (green contour) for each long-axis cine view (**panels B, E**). Inward displacement was quantified by measuring the change in position of the endocardial tracking points from the corresponding centreline of the LV during systole, expressed as a percentage (**panel B**). LV segmental inward displacement is shown in parametric format, using a 17-segment model (**panel C, F**). Segments which shorten during systole are represented in red, whilst dyskinetic segments are shown in blue

CMR data analysis was performed offline using Medis Suite software (Medis Medical Imaging BV, Leiden, The Netherlands). LV endocardial and epicardial contours were drawn automatically using the AutoQ function on both short-axis and long-axis cine images. Subsequently, LV contours were automatically propagated in all frames throughout the entire cardiac cycle. Papillary muscles were considered part of the LV cavity, and epicardial adipose tissue was excluded from the region of interest. In case of inadequate automated tracing, the endocardial border was manually adjusted. LV end-diastolic volume, LV end-systolic volume, and LVEF were calculated from short axis reconstructions (QMass 8,1, Medis Suite software). LVGRS was calculated from short axis views and LVGLS was calculated from the long-axis views (2, 3, and 4-chamber) with commercially-available software (QStrain 4.1, Medis Suite software) utilizing FT-CMR. LV InD was quantified by measuring the displacement of (automatically) tracked endocardial points towards the centerline of the LV during systole from long-axis images (2, 3, and 4-chamber). The centerline was located along the LV long axis with a position that varied between one-half and two-thirds of the base-apex distance and was determined automatically by the software (Fig. 1). The endocardial displacement was expressed as a percentage, after normalization to the distance at end-diastole between the endocardial points and the corresponding LV centerline [14]. The American Heart Association 17-segment model was used to evaluate segmental LV InD,[21] and the LV InD was calculated as the mean value of the end-systolic LV InD for each myocardial segment (Fig. 1).

LGE (scar) was assessed on the basis of signal intensity (SI) [20]. The myocardial segment with the most dense scar was visually identified and used to define the reference maximum SI by drawing a region of interest. LV myocardium with a SI ≥ 35% of the maximal SI was defined as scar tissue on automated analysis, and expressed as a percentage of LV volume [20, 22].

### Follow-up and endpoints

The study endpoint was a composite of all-cause mortality, heart failure hospitalization and arrhythmic events. Data on mortality and heart failure hospitalization were obtained from the departmental cardiology information system (EPD Vision, version 12.5.4, Leiden University Medical Centre, Leiden, The Netherlands) which is linked to the governmental death registry database. Arrhythmic events were defined as documented ventricular fibrillation, sustained ventricular tachycardia lasting > 30 s, appropriate ICD therapy or resuscitation after cardiac arrest. The occurrence of ICD therapy was assessed by device interrogation.

### Statistical analysis

Continuous data were presented as mean ± standard deviation when normally distributed or median and interquartile range (IQR) when not normally distributed. Categorical variables were expressed as frequencies and percentages. The study population was dichotomized according to the median value of LV InD. LV InD groups were compared using the independent samples Student *t*-test for continuous variables with a normal distribution, or the Mann-Whitney *U*-test for non-normally distributed continuous variables, whereas categorical variables were compared using the Pearson chi-square test or Fisher’s exact test, as appropriate. Kaplan-Meier survival analysis was used to estimate the event-free survival rate for the composite endpoint. Bivariate and multivariate Cox regression analyses were performed to evaluate the association between baseline variables and the composite endpoint. Significant variables on bivariate analysis (P-value < 0.05) were included in the multivariate regression analysis. To avoid multicollinearity, variables with a variance inflation factor exceeding 10 were excluded from the multivariate analysis. To investigate the incremental prognostic value of LV InD over various clinical and imaging parameters, including age, sex, LVEF, scar burden, LVGRS and LVGLS, the change in the likelihood ratio (LR) chi-square value for nested models was calculated. Fourteen random patients were selected for the evaluation of intra- and inter-observer variability of LV InD, using intraclass correlation coefficients for absolute agreement of average measures. Moreover, intra- and interobserver agreement for LV InD was evaluated by Bland-Altmann analysis. Two-sided P-values < 0.05 were considered significant. The statistical analyses were performed using SPSS version 25 (SPSS, Inc., Chicago, IL, USA) and R (version 4.1.1; R Foundation for Statistical Computing, Vienna, Austria).

## Results

### Study population: clinical and imaging characteristics

A total of 111 patients (mean age 57 ± 10 years, 86% male) with a history of myocardial infarction, who underwent LGE-CMR imaging, were included based on inclusion and exclusion criteria. The median time from the index acute MI to CMR was 81 [IQR: 53–168] days. The typical indications for CMR were to assess myocardial viability (n = 92, 83%). Other indications were for the assessment of ventricular function (n = 14, 13%), diagnosis of other etiologies of LV dysfunction (n = 3, 3%), or assessment of myocardial ischemia by stress CMR (n = 2, 2%). The majority of patients were classified as ST-segment elevation myocardial infarction (STEMI)(n = 101, 94%), while the remainder had non-STEMI (n = 7, 6%). For the measurement of LV InD, 9 (10%) of patients required manual contour adjustment. Amongst cardiovascular risk factors, diabetes mellitus and chronic kidney disease were seen more commonly in patients with LV InD < 23.0. The use of aspirin, angiotensin-converting enzyme inhibitors (or angiotensin receptor blockers), beta-blockers and statins exceeded 95% in all groups (Table 1). ICD or CRT implantation was performed in 22 (19.8%) patients after CMR.

**Table 1:** Baseline patient characteristics

Variables	Overall population (n = 111)	LV InD < 23.0 (n = 55)	LV InD ≥ 23.0 (n = 56)	P-value
Age	57.4 ± 9.9	59.4 ± 10.6	55.5 ± 8.9	0.059
Sex, male, n (%)	96 (86%)	48 (87%)	48 (86%)	0.810
Arterial hypertension, n (%)	46 (41%)	24 (44%)	22 (39%)	0.642
Hyperlipidaemia, n (%)	31 (28%)	20 (36%)	11 (20%)	0.050
Diabetes mellitus, n (%)	14 (13%)	11 (20%)	3 (5%)	0.024
Chronic kidney disease, n (%)	6 (6%)	6 (11%)	0 (0%)	0.012
**Medical therapy**				
Aspirin, n (%)	106 (95%)	53 (96%)	53 (95%)	>0.999
ACEI/ARB, n (%)	110 (99%)	54 (98%)	56 (100%)	0.495
Beta-blocker, n (%)	107 (96%)	52 (95%)	55 (98%)	0.364
Statin, n (%)	111 (100%)	55 (100%)	56 (100%)	>0.999
**CMR parameters**				
LVEF (%)	44.8 ± 14.5	34,5 ± 11.9	55.3 ± 8.0	< 0.001
LVEDV (ml)	214.4 ± 66.8	255.3 ± 65.3	172.8 ± 35.2	< 0.001
LVESV (ml)	125.9 ± 71.0	172.5 ± 71.2	78.5 ± 23.9	< 0.001
LVGRS (%)	38.3 ± 21.4	48.1 ± 20.8	28.3 ± 17.1	< 0.001
LVGLS (%)	16.1 ± 6.5	10.9 ± 4.2	21.2 ± 3.8	< 0.001
LV inward displacement (%)	22.1 ± 7.7	15.6 ± 4.4	28.6 ± 3.9	< 0.001
LV scar burden (%)	15.4 [5.2–22.7]	22.7 [13.8–31.9]	7.2 [0.9–12.4]	< 0.001

Values are expressed as mean ± SD or median [25-75%]. ACEI, angiotensin-converting enzyme inhibitor; ARB, angiotensin receptor blocker; InD, inward displacement; LVEDV, left ventricular end-diastolic volume; LVEF, left ventricular ejection fraction; LVESV, left ventricular end-systolic volume; LVGRS, left ventricular global radial strain; LVGLS, left ventricular global longitudinal strain

The mean LVEF was 50.4 ± 13.4% and 47 (41%) patients had an LVEF < 50%. The LV end-diastolic and end-systolic volumes were significantly larger in the group with LV InD < 23.0, while the LVEF, LVGLS and LVGRS were more impaired in this group. On LGE-CMR, LV scar tissue was present in 97 (89%) patients (transmural in 40 patients and subendocardial in 57 patients). The median LV scar volume was 15.4% [IQR5.2-22.7%], and the burden of post-infarction scar was significantly greater in patients with LV InD < 23.0. LV scar burden was also greater in patients with transmural scar than in those with subendocardial scar (17.0 ± 9.7% vs. 8.5 ± 7.5%, P < 0.001). LV InD (17.3 ± 7.2% vs. 23.7 ± 6.1%, P < 0.001) and LVGLS (12.3 ± 6.4% vs. 17.4 ± 5.4%, P < 0.001) were lower in patients with transmural scar than in those with subendocardial scar only.

### Survival analysis and incremental value of LV InD

**Fig. 2:**
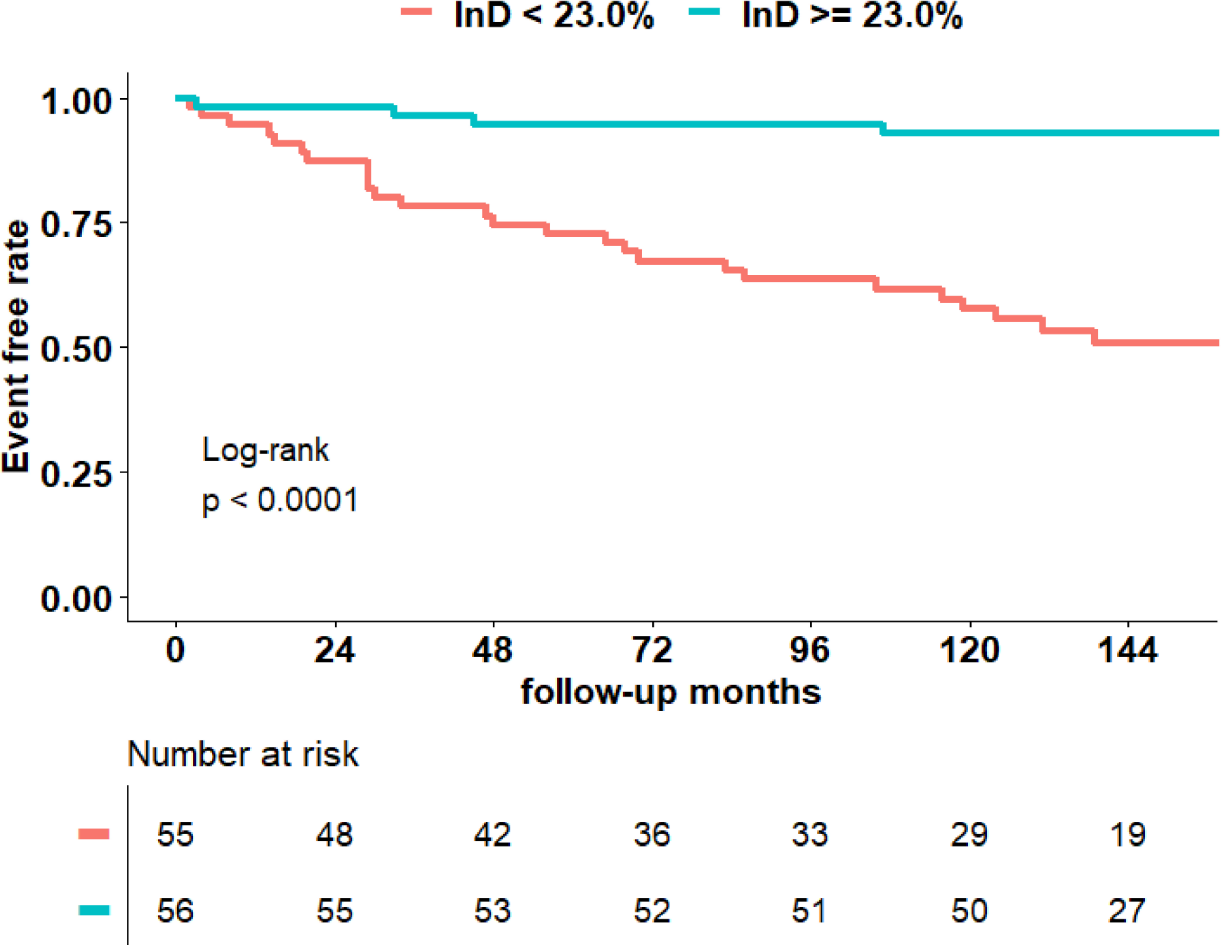
Kaplan-Meier curve for the composite endpoint of survival, heart failure hospitalization and arrhythmic events, using a threshold of 23% for left ventricular inward displacement A significantly greater event-free survival was observed in patients with left ventricular inward displacement InD ≥ 23%, compared to those with a InD < 23%.

Over a median follow-up of 142 (IQR 107–159) months, 31 (27.9%) events occurred (12 all-cause deaths, 5 heart failure hospitalizations and 14 arrhythmic events). Arrhythmic events included 1 episode of ventricular fibrillation, 2 sustained ventricular tachycardias, 2 resuscitations after cardiac arrest, and 9 appropriate ICD therapies. Patients with more impaired LV InD (< 23.0) experienced a significantly lower event-free survival (P < 0.001) (Fig. 2). Age, LVEF, LV scar burden, LVGRS, LVGLS and LV InD were associated with the combined endpoint of all-cause mortality, heart failure hospitalization and arrhythmic events on bivariate Cox regression analysis. On multivariate analysis, age and LV InD (P = 0.010) remained independently associated with the composite endpoint (Table 2). LVEF, LVGRS, and LVGLS were excluded from the multivariate model due to multicollinearity.

**Table 2:** Cox regression analysis for the composite endpoint

	Bivariate analysis				Multivariable analysis		
**Variables**	**HR**	**95% CI**	**P-value**	**HR**		**95% CI**	**P-value**
Age	1.06	1.02–1.11	0.002	1.04		1.00-1.09	0.036
Sex, male	0.63	0.26–1.54	0.312	-		-	-
Arterial hypertension	1.28	0.63–2.60	0.486	-		-	-
Hyperlipidaemia	1.66	0.81–3.43	0.168	-		-	-
Diabetes mellitus	1.01	0.35–2.90	0.982	-		-	-
Chronic kidney disease	2.29	0.69–7.53	0.174	-		-	-
Aspirin	0.76	0.18–3.20	0.711	-		-	-
ACEI/ARB	0.20	0.03–1.50	0.118	-		-	-
Beta-blocker	0.59	0.14–2.45	0.464	-		-	-
LVEF (%)	0.95	0.93–0.97	< 0.001	-		-	-
LVGRS (%)	0.96	0.94–0.98	< 0.001	-		-	-
LVGLS (%)	0.88	0.83–0.93	< 0.001	-		-	-
LV inward displacement (%)	0.89	0.84–0.93	< 0.001	0.90		0.84–0.98	0.010
LV scar burden (%)	1.04	1.02–1.06	0.001	1.00		0.95–1.06	0.959

ACEI, angiotensin-converting enzyme inhibitor; ARB, angiotensin receptor blocker; CI, confidence interval, HR, hazard ratio, LVEDV, left ventricular end-diastolic volume; LVEF, left ventricular ejection fraction; LVESV, left ventricular end-systolic volume; LVGRS, left ventricular global radial strain; LVGLS, left ventricular global longitudinal strain

**Fig. 3:**
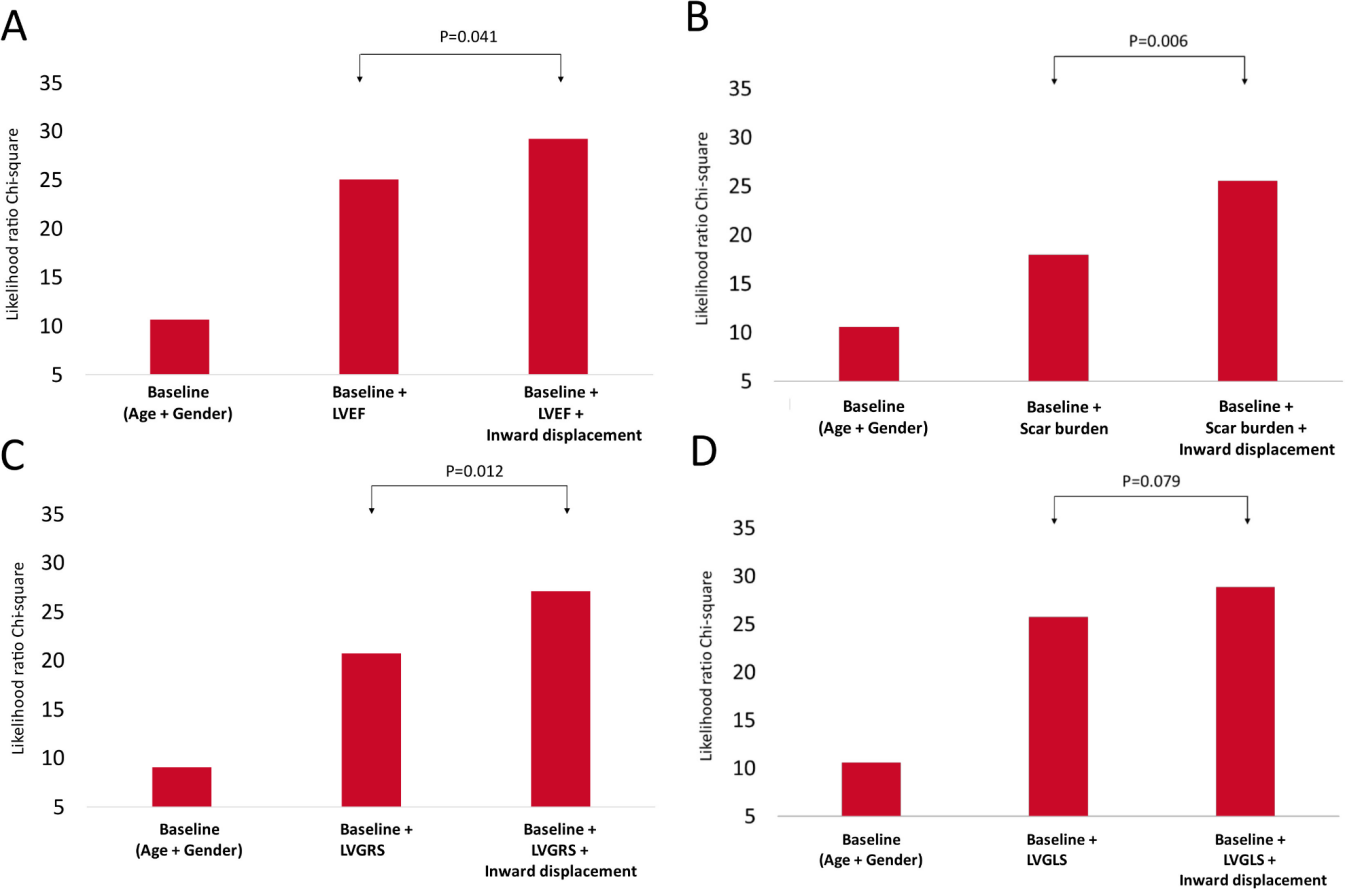
Likelihood ratio test for the incremental value of left ventricular inward displacement for the composite endpoint of survival, heart failure hospitalization and arrhythmic events LV InD adds significant prognostic value to models including baseline clinical parameters (age and sex), LVEF (**panel A**), LV scar burden (**panel B**) and LVGRS (**panel C**). No significant increase in the chi-square value was observed when LV InD was added to LVGLS (**panel D**). InD, inward displacement; LVEF, left ventricular ejection fraction; LVGRS, left ventricular global radial strain; LVGLS, left ventricular global longitudinal strain

LR nested analysis demonstrated incremental prognostic value when adding LV InD to models including LVEF (P = 0.041), LV scar burden (P = 0.009) and LVGRS (P = 0.012). LV InD did not add significant incremental predictive value to a model which included LVGLS (P = 0.079)(Fig. 3).

### Reproducibility of CMR-derived LV InD

**Fig. 4:**
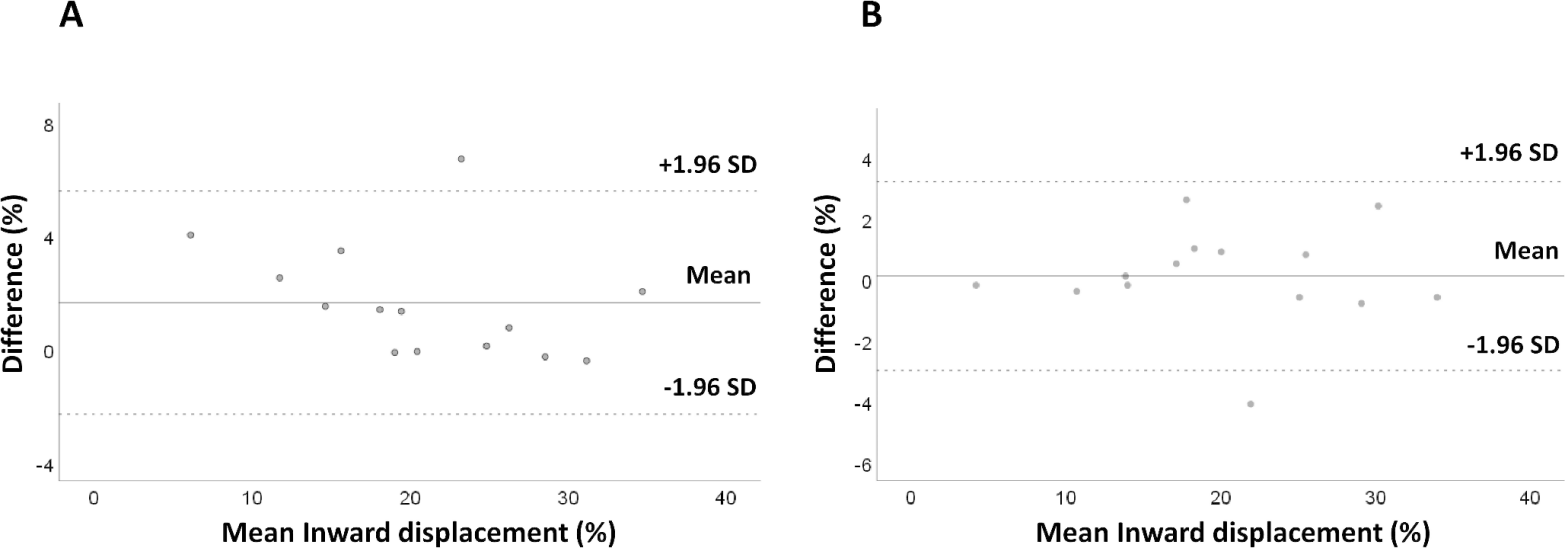
Intra- (**A**) and inter-observer (**B**) agreement for left ventricular inward displacement, assessed by Bland-Altman analysis SD: standard deviation

The intra-observer agreement for LV InD was good, with a mean difference of 1.6 ± 4.0% between repeated measures (Fig. 4A). The intraclass correlation coefficient for intra-observer comparison was 0.99. Similarly, good agreement was noted between measurements obtained by different observers, with a mean difference of -0.5 ± 3.2% and an intraclass correlation coefficient of 0.99 (Fig. 4B).

## Discussion

In patients with IHD and previous myocardial infarction, FT-CMR-derived LV InD was independently associated with long-term outcomes. Furthermore, LV InD provided incremental prognostic value to LVEF, LVGRS and LV scar burden, but not to LVGLS.

LVEF has been the mainstay of LV systolic function quantification for six decades, and it is recommended to measure LVEF in all patients with myocardial infarction [3]. LVEF has also robust data to support its use as prognostic marker in patients who have experienced a myocardial infarction [4, 23]. LVEF, however, is subject to a number of important limitations, e.g. its load-dependency, insensitivity to mild dysfunction, its dependence on LV geometry (e.g. hypertrophy) and dyssynchrony (e.g. left bundle branch block) [6, 24, 25]. Deformation imaging is more sensitive to subtle changes in LV systolic dysfunction, and also less load-dependent than LVEF. LVGLS has been found to provide incremental prognostic value over LVEF for patients with IHD [3]. Strain can be measured not only from speckle tracking strain echocardiography, but also from FT-CMR, which has emerged as a superior parameter of LV systolic function for post-infarct risk stratification when compared to LVEF [12, 26]. In a multicentre study including > 1200 patients with history of myocardial infarction, LVGLS, LV global circumferential strain, and LVGRS derived from FT-CMR, were all significantly associated with outcome. In particular, LVGLS demonstrated additive predictive value for all-cause mortality over and above LVEF, and it has become the preferred deformation parameter in clinical practice due to its reproducibility [12].

The presence and extent of LGE are predictive of ventricular arrhythmias and sudden cardiac death in patients with and previous myocardial infarction, since the border (transition) zone between normal myocardium and scar tissue creates the substrate for an electrophysiologic re-entry circuit to be established [27, 28]. While extensive scar tissue (represented by LGE on CMR) impacts on LV systolic function, it nevertheless represents an independent risk factor for long-term outcomes in these patients due to its association with lethal arrhythmias. LV InD demonstrated additive predictive value over post-infarction scar, when imaged by LGE CMR.

LV deformation parameters, such as LVGLS or LVGRS, while being sensitive to detect global systolic LV function, are more limited in their ability to identify regional LV systolic impairment. In addition, these strain parameters reflect LV deformation in one direction only, while in reality, LV contraction comprises circumferential, radial, and longitudinal changes. Systolic LV wall motion can be described by the inward motion of the endocardium towards the centerline of the ventricle, which potentially overcomes the limitations of existing strain parameters, i.e.: (1) it can reflect multidirection (radial and longitudinal) LV wall motion and (2) it may be more sensitive for the identification of regional systolic function [14]. While systolic wall motion analysis has been used for four decades, with both echocardiography and CMR, it has not been based on a solid analytical model of endocardial motion. FT-CMR, despite being most commonly used for deformation analysis, has also allowed a paradigm shift to occur in LV systolic wall motion analysis, due to the highly accurate tracking of the endocardial border. This has resulted in the emergence of a novel parameter of LV wall motion, namely LV InD, which is not based merely on visual analysis. In the current study, LV InD provided incremental prognostic value over LVEF and LVGRS. These results might be explained by the fact that LV InD affect not only radial motion as indicated by LVGRS, but also LV wall motion in multiple directions. Conversely, LV InD was not clearly shown to be superior over LVGLS in the present study. LVGLS is highly sensitive to wall motion abnormalities in patients with IHD, since it primarily affects longitudinal, subendocardial myocardial fibres [3, 29]. The prognostic value of LV InD might, therefore, be limited in comparison to LVGLS, specifically in patients with IHD. This does not detract from the fact that LV InD may provide a superior measure of regional LV function over LVGLS, while its use in non-ischemic cardiomyopathies, where the subendocardial fibres are not necessarily the most severely affected, remains to be elucidated.

Post-infarct risk stratification and the decision to implant an ICD in patients with IHD is currently mainly based on the LVEF. However, the use of LVEF to predict sudden cardiac death in these patients, is neither sensitive nor specific, and a clinical need exists for a refinement in risk stratification [6]. LV InD provides incremental prognostic value over established biomarkers (i.e. LVEF, LVGRS and scar burden on LGE-CMR). Moreover, LV InD can be rapidly and automatically measured with a high degree of reproducibility by commercially available software without additional risk or cost. Additionally, it is an intuitive measure to interpret. As a results, this novel imaging parameter is a prime candidate for enhancing post-infarct risk stratification in clinical practice.

### Limitations

This study is subject to the limitations of its retrospective, single-centre, observational design. Since only patients who underwent clinically-indicated CMR were included, selection bias cannot be excluded, and the limited number of patients precludes subgroup analysis, e.g. in patients with reduced versus preserved LVEF. In addition, no distinction could be drawn between all-cause and cardiac mortality. Another limitation could be that LV InD is only available from a single vendor.

## Conclusion

In conclusion, LV InD, measured with FT-CMR, was independently associated with outcomes in patients with prior myocardial infarction when correcting for cardiovascular risk factors. LV InD also provided incremental prognostic value over LVEF and LVGRS. Accordingly, LV InD holds promise as a pragmatic imaging biomarker for post-infarct risk stratification.
